# An Aging Nursing Workforce: Thematic Analysis from the Nurstory Project

**DOI:** 10.1093/geroni/igab046.2292

**Published:** 2021-12-17

**Authors:** Raeann LeBlanc

**Affiliations:** University of Massachusetts, Amherst, Royalston, Massachusetts, United States

## Abstract

Background: Nursing in the United States of America is an aging workforce. This study sought to better understand the lived experience of aging nurses. Because nurses work in systems where other forms of interpersonal power dynamics may influence internalized and external stereotype an approach based on intersectional theory was applied. Methods: A qualitative thematic narrative analysis of an existing data set of first-person digital stories in the Nurstory project, authored by a group of nurses, was the data source. An emergent coding method was applied. The collection of five digital stories were analyzed. Results: All stories were first person accounts of experiences that represented their internalized reflections and elements of ageism in how their age interacted with their work environment. Dominant themes included: 1) Role constriction 2) Strength 3) Tired and (re)Tired 4) Age perceived and 5)Loneliness. Conclusions: These aging nursing stories add to the contextual layers of the aging healthcare workplace and aging nursing workforce. These individual experiences offer a nuanced understanding of the internalized responses to aging and ageism. These stories highlight socially constructed and socially reinforced attitudes that are complicated by the personal and occupational expectations of nurse’s work, their role and embedded hierarchies in healthcare. Stories such as these are important individual and collective indicators of lived experiences that offer a deeper understanding into the intersections of social identity and aging, that when listened to, can offer insight and a way forward in addressing the stereotype, discrimination and social inequities of ageism.

